# Case Report: Genetically primed hyperinflammation: cytomegalovirus-triggered HLH-like syndrome in an adolescent with a gain-of-function STING1 (p.Arg281Trp) variant with novel autosomal dominant inheritance and atypical presentation

**DOI:** 10.3389/fimmu.2026.1801755

**Published:** 2026-06-11

**Authors:** Ehab Abdelbadeeh Hassan Hammad, Tariq Zulfiquar Ali, Dieter Clemens Broering, Hassan Ali Aleid, Quaid Johar Nadri, Rehab Salah Fathy Nasr

**Affiliations:** 1 Adult Transplant Nephrology, Kidney and Pancreas Health Centre, King Faisal Specialist Hospital and Research Centre, Riyadh, Saudi Arabia; 2 Department of Internal Medicine and Nephrology, Faculty of Medicine, Fayoum University, Fayoum, Egypt; 3 Organ Transplant Centre of Excellence, Organ Transplant Centre of Excellence, King Faisal Specialist Hospital and Research Centre, Riyadh, Saudi Arabia; 4 Department of Internal Medicine and Nephrology, Faculty of Medicine, Benha University, Benha, Egypt

**Keywords:** autosomal dominant, CMV infection, HLH-Like, interferopathy, p. Arg281Trp variant, SAVI, STING1

## Abstract

We report the case of an 18-year-old previously healthy female who developed acute liver and kidney injuries with cytopenia and hyperinflammatory markers following cytomegalovirus (CMV) infection. Despite not meeting the hemophagocytic lymphohistiocytosis (HLH)-2004 or H-score criteria, her clinical and biochemical profiles suggested an HLH-like syndrome. Whole-exome sequencing revealed a heterozygous dominant stimulator of interferon genes (STING1) (c.841C>T; p. Arg281Trp) mutation. Although the patient lacked classic STING-associated vasculopathy with onset in infancy (SAVI)-associated skin or lung manifestations, immunological profiling revealed an inverted cluster of differentiation (CD)4/CD8 ratio and absolute CD4+ lymphopenia, supporting a state of underlying immune dysregulation that likely predisposed the patient to severe CMV infection. The patient showed partial clinical and biochemical improvements following antiviral and corticosteroid therapy, supporting our hypothesis of a genetically primed infection-triggered hyper-inflammatory response. This case broadens the STING1 disease spectrum and emphasizes the importance of genomic evaluation of unexplained hyperinflammation, even in apparently immunocompetent hosts. Conclusion: This case highlights a novel STING1 (p. Arg281Trp) gain-of-function mutation with a previously unreported autosomal dominant inheritance pattern identified in both the patient and her asymptomatic father, suggesting incomplete penetrance and variable expression rather than a fully penetrant autosomal dominant pattern. The patient’s presentation was atypical compared with classical SAVI, manifesting as a CMV-triggered HLH-like syndrome with acute renal and liver cell injury in an adolescent with no prior evidence of immune deficiency or family history of genetic disease. Notably, this presentation lacked the characteristic cutaneous and pulmonary involvement. This case highlights a distinct phenotypic spectrum and expands our understanding of STING1-related interferonopathies.

## Background

Stimulator of interferon genes (STING)-associated vasculopathy with onset in infancy (SAVI) is a rare autoinflammatory disorder caused by gain-of-function mutations in the transmembrane protein 173 (TMEM173) gene, which encodes the STING protein. This mutation results in the constitutive activation of the type I interferon pathway, leading to systemic inflammation and tissue injury. Classic presentations include early onset cutaneous vasculopathy, acral necrosis, and interstitial lung disease, which are often accompanied by recurrent fever and growth impairment (1–4).

However, emerging evidence indicates significant phenotypic variability, as some patients with SAVI exhibit incomplete or atypical manifestations. Reports of cases lacking the full spectrum of cutaneous or pulmonary involvement suggest that certain mutations may result in milder or more organ-limited phenotypes. Such atypical presentations can delay diagnosis and complicate management, particularly in the absence of a family history of genetic or immunodeficiency disorders (5–11).

Although SAVI typically presents during early childhood, this case highlights an adolescent-onset presentation, thus expanding the recognized age spectrum and emphasizing the importance of considering STING1-related interferonopathies in pediatric and adolescent patients presenting with unexplained hyperinflammation. Recognition of these atypical forms is essential, as timely molecular diagnosis enables early initiation of targeted therapies, such as JAK inhibitors, to modulate the overactive interferon signaling pathway. Documenting these non-classical presentations contributes to a broader understanding of the clinical spectrum of SAVI and may help inform future genotype–phenotype correlations (3, 12–17).

## Case presentation

**Figure 1:**
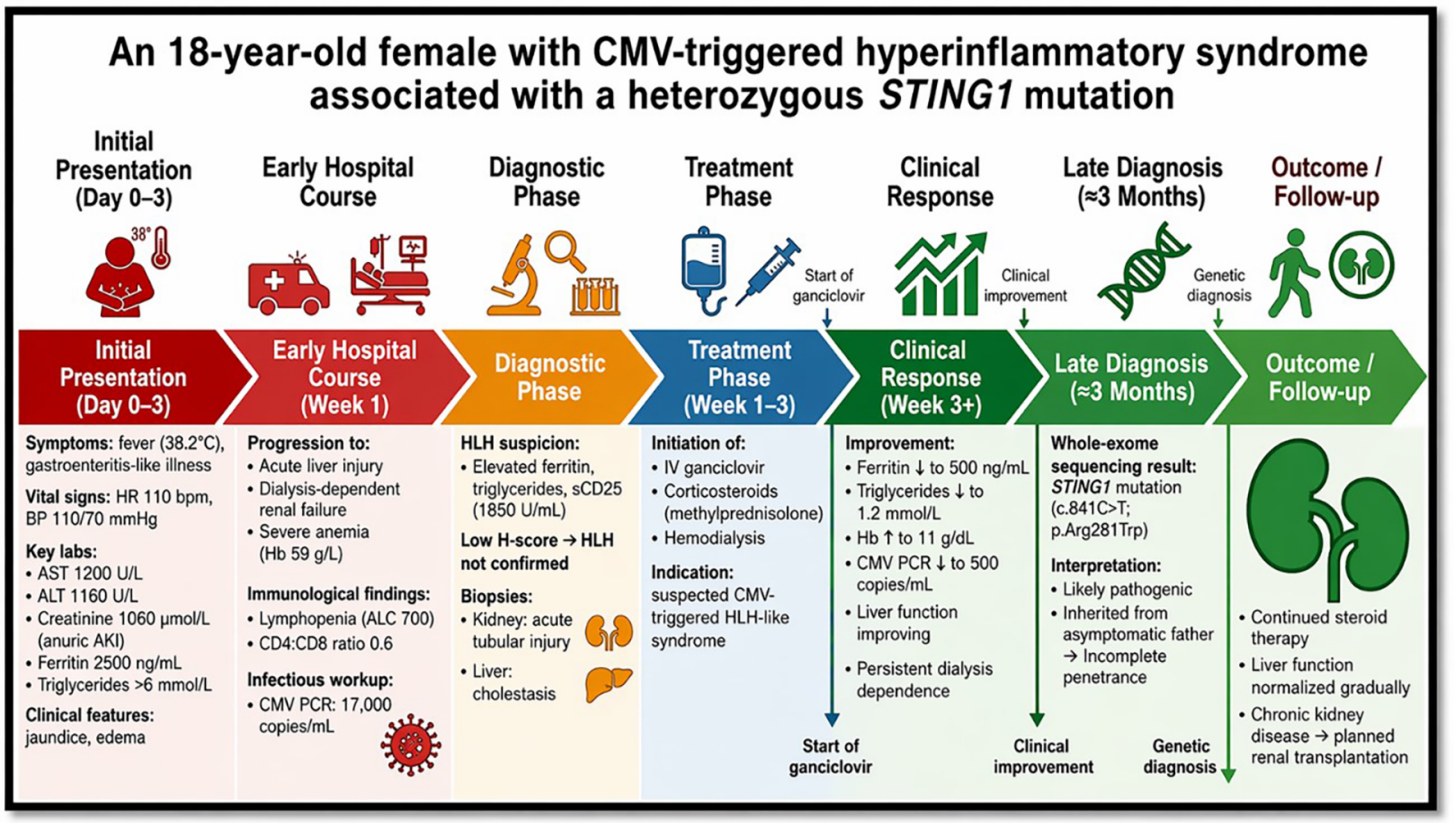
Timeline of disease progression, management, and outcomes.

An 18-year-old previously healthy female initially presented with a 3-day history of acute gastroenteritis-like symptoms that rapidly progressed to severe liver and kidney injuries. Notable vital signs at presentation included high temperature and tachycardia (**Table 1**; **Figure 1**).

**Table 1:** Clinical and laboratory findings of the patient at presentation and during follow-up.

Parameter	At presentation	1 week	4 weeks	3 months	6 months	Reference range
Vital signs						
Temperature (°C)	38.2	38.6	37	37.4	36.5	36.5–37.5
Heart rate (bpm)	110	105	108	95	100	60–100
Blood pressure (mmHg)	110/70	130/80	125/85	128/75	140/86	≤120/80
SpO_2_ (% RA)	98	96	95	99	97	≥94
Hematological parameters						
Hemoglobin (g/L)	59 (normocytic anemia)	75 (post transfusion)	111	105	110 (EPO therapy)	120–160
Platelets (×10^9/L^)	250	203	300	190	258	**1509/L)**
Reticulocyte count (×10^9/L^)	20	18	30	65	50	25–85
Iron saturation (%)	60	—	—	30		20–50
LDH (U/L)	328	350	280	216	—	140-280
Other investigations (haptoglobin, indirect bilirubin, Coombs test, peripheral blood smear [schizocytes], folate, and vitamin B12 levels, Hb electrophoresis)	Normal	—	Normal	—	—	—
Renal function						
Creatinine (µmol/L)	1050	Dialysis-dependent	Dialysis-dependent	Dialysis-dependent	Dialysis-dependent	45–90
ACR (g/g)	1	0.5	—			<0.03
Sodium (mmol/L)	120	126	130	135	130	135–145
Liver function						
AST/ALT (U/L)	1200/1150	1700/1400	1005/954	304/275	26/36	<40
Direct bilirubin (µmol/L)	250	300	100	50	4	<5
ALP (U/L)	520	700	300	140	150	46–122
GGT (U/L)	200	233	120	65	35	5-36
PT/PTT (seconds)	14.4/42	15.4/40	14.6/32	13.3/35	13/38	11–13.5/25–40
HFE mutation and ceruloplasmin	—	—	Normal			—
Inflammatory markers						
Ferritin (ng/mL)	2500	2900	500	400	530	15–150
CRP (mg/L)	150	170	50	32	18	<5
Procalcitonin (ng/ml)	0.03	0.05	0.02	0.06	0.04	<0.05
sCD25 (U/mL)	1850	—	—	1600	—	158–623
TG/cholesterol (mmol/L)	6/15	7/16	1.2/4	1.5/4.8	—	<1.7/ <5.2
Immunological workup						
Absolute lymphocyte count (cells/µL)	—	700	—	1238		1500–4300
CD4/CD8 ratio	—	0.6	—	0.58		0.8–2.4
IgG (g/L)	—	5	5.9	6.5	—	6.6–15.3
Other Ig: IgA, IgM, pneumococcal cap polysaccharide Ab, Tetanus toxoid Ab	Normal	—	—	Normal	—	
Autoimmune panel (ANA, dsDNA, RF, ANCA, anti-GBM, anti-CCP, Scl-70, LKM Ab, ASMA, AMA, anti-Ro/La, and C3, C4)	Negative	—	Negative	—	—	—
Infectious workup						
CMV PCR (copies/mL)	—	17,000	500	Undetectable	Undetectable	Negative
Other septic work-up (Viral PCRs [HCV, HIV, HBV, HEV, HAV, CMV, EBV, Parvo virus, COVID], Legionella Ag in urine, Leptospira Ab, Toxoplasma Ab, and blood/stool/urine cultures)	Negative	—	—	—	—	—

ACR, albumin-creatinine ratio; ALP, alkaline phosphatase; ALT, alanine aminotransferase; AST, aspartate aminotransferase; CMV, cytomegalovirus; CRP, C-reactive protein; Ig G, immunoglobulin G; sCD25, soluble interleukin-2 receptor; RA, room air; EPO, erythropoietin; TG, triglycerides; LDH, lactate dehydrogenase; PT, prothrombin time; PTT, partial thromboplastin time.

Initial laboratory evaluation (**Table 1**) revealed severe mixed hepatocellular and cholestatic liver injury, along with anuric acute kidney injury (AKI) requiring urgent initiation of hemodialysis. Her clinical course was further complicated by recurrent unexplained severe normocytic normochromic anemia, necessitating frequent transfusions. Physical examination results were otherwise unremarkable except for jaundice and lower extremity edema.

Subsequent laboratory investigations (**Table 1**) revealed marked hyperferritinemia, hypertriglyceridemia, hypercholesterolemia, hyponatremia, and elevated iron saturation. The anemia workup, including haptoglobin, reticulocyte count, indirect bilirubin, Coombs test, peripheral blood smear, and measurements of lactate dehydrogenase (LDH), iron, folate, and vitamin B_12_ levels, was largely unremarkable, except for reticulocytopenia and iron overload. HFE genetic testing yielded negative results, thereby excluding hereditary hemochromatosis as a cause of iron overload.

Subsequently, an extensive autoimmune workup was performed. Autoimmune markers—including antinuclear antibody (ANA), anti–double stranded DNA (dsDNA), rheumatoid factor (RF), antineutrophil cytoplasmic antibodies (ANCA), anti-glomerular basement membrane (anti-GBM), anti-cyclic citrullinated peptide (anti-CCP), anti-topoisomerase I (anti-Scl-70), liver kidney microsomal antibody (LKM), anti-smooth muscle antibody (ASMA), anti-mitochondrial antibody (AMA), anti-Sjögren’s-syndrome-related antigen A (anti-SSA/Ro), anti-Sjögren’s-syndrome-related antigen B (anti-SSB/La), and complement C3 and C4—were all unremarkable. Familial hyperlipidemia was excluded through genetic testing.

Infectious and septic workups, including polymerase chain reaction (PCR) assays for hepatitis C virus (HCV), human immunodeficiency virus (HIV), hepatitis B virus (HBV), hepatitis E virus (HEV), hepatitis A virus (HAV), cytomegalovirus (CMV), Epstein–Barr virus (EBV), parvovirus, and coronavirus disease 2019 (COVID-19), along with investigations including *Legionella* antigen in urine, *Leptospira* antibody, *Toxoplasma* antibody, and blood, stool, and urine cultures, were all negative except for high-level CMV viremia (PCR: 17,000 copies/mL).

Immunological assessment revealed lymphopenia and hypogammaglobulinemia (IgG 5 g/L). Inflammatory markers were notable, with an elevated C-reactive protein (CRP) level of 150 mg/L and normal procalcitonin levels (**Table 1**).

Abdominal ultrasound, chest radiography, magnetic resonance cholangiopancreatography (MRCP), and positron emission tomography-computed tomography (PET-CT) were unremarkable except for evidence of iron overload, ascites, and pleural effusion. Given the constellation of findings, kidney and liver biopsies were performed.

Renal biopsy was performed under light microscopy with hematoxylin and eosin (H&E) and Masson’s trichrome staining. The results revealed moderate acute tubular injury with mild interstitial edema and minimal mononuclear inflammatory infiltrate associated with focal mild tubulitis, consistent with acute interstitial nephritis. The glomeruli demonstrated preserved architecture without global or segmental sclerosis or significant mesangial abnormalities. No evidence of interstitial fibrosis or tubular atrophy was found, and the vasculature was unremarkable. Immunofluorescence was negative for immune deposits, and electron microscopy revealed normal glomerular basement membranes with focal podocyte foot process effacement. Viral cytopathic changes or CMV immunostaining were not detected in the renal tissue.

Liver biopsy demonstrated zone 3 hepatocellular and canalicular cholestasis without significant portal or lobular inflammation. No evidence of chronic liver disease, bile duct injury, steatosis, or iron accumulation was found. CMV staining of biopsy specimens was negative.

Given the presence of marked hyperinflammation (elevated ferritin and CRP), hypertriglyceridemia, and multi-organ failure (hepatic, renal, and hematologic), combined with otherwise negative malignancy, autoimmune, and infectious workups, excluding CMV with negative immunostaining in tissue biopsies, direct CMV nephritis and CMV hepatitis were excluded. Consequently, a predominantly inflammation-mediated mechanism of injury and a state of immune dysregulation were suspected.

The possibility of hemophagocytic lymphohistiocytosis (HLH) was considered. Soluble interleukin-2 receptor (sIL-2R/sCD25) levels were markedly elevated at 1850 U/mL. However, owing to a low H-score (3%–5%), a bone marrow biopsy was deferred. The patient was treated with intravenous ganciclovir and corticosteroids because of strong suspicion of a CMV-triggered HLH-like inflammatory syndrome, along with supportive transfusions and ongoing dialysis. Following the exclusion of acquired etiologies, whole-exome sequencing was performed to identify any underlying genetic causes of combined immunodeficiency and hyperinflammation.

After three weeks of antiviral and corticosteroid therapy, the patient demonstrated marked clinical and biochemical improvement, with inflammatory markers, triglycerides, and hemoglobin levels normalizing without further transfusion requirements, along with a significant decline in CMV viral load and a mild improvement in the liver profile (**Table 1**).

**Figure 2:**
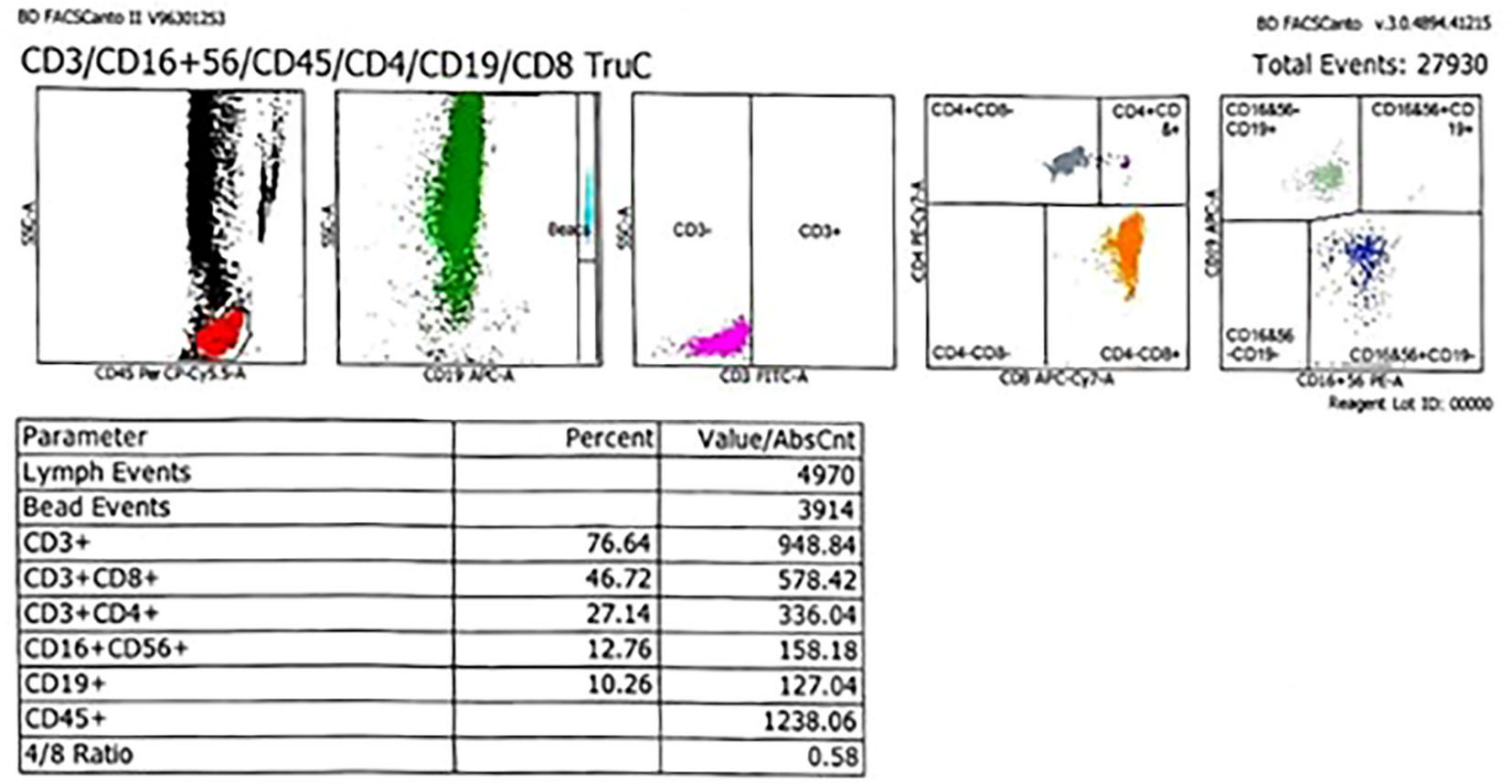
Flow cytometric immunophenotyping was performed 3 months after presentation using the BD Multitest™ 6-color TBNK reagent with BD Trucount™ technology (single-platform assay). Lymphocytes were initially identified using CD45 versus side scatter gating. The second plot, labeled CD19 in the exported report, was used for TruCount bead identification for absolute cell counting and was not used as a sequential parent gate for T-cell analysis. T-cell (CD3+), B-cell (CD19+), and NK-cell (CD16/CD56+) populations were subsequently identified according to the standard assay protocol, with CD4/CD8 subset analysis performed within CD3+ T cells.

At the three-month follow-up, a further marked improvement in liver function was observed. Subsequent lymphocyte subset analysis via flow cytometry demonstrated a mild upward trend in the absolute lymphocyte count; however, the cluster of differentiation CD4/CD8 ratio remained significantly inverted, consistent with chronic T-cell activation and exhaustion (**Table 1**; **Figure 2**). Despite these improvements, the patient remained dialysis-dependent.

Whole-exome sequencing results were received late and revealed a heterozygous dominant STING1 c.841C>T (p. Arg281Trp) mutation, which was reported as likely pathogenic. This specific p. Arg281Trp variant was previously characterized as a gain-of-function mutation that induces constitutive STING activation and high interferon-stimulated gene (ISG) expression.

Whole-exome sequencing was performed in an accredited clinical laboratory using a validated pipeline. Genomic DNA was prepared using the Illumina DNA PCR-Free Prep kit and sequenced on the Illumina NovaSeq 6000 platform. Sequencing reads were aligned to the Genome Reference Consortium Human Build 38 (GRCh38) reference genome using a locally installed Illumina Dynamic Read Analysis for GENomics (DRAGEN) pipeline. Mean sequencing coverage exceeded 30×, with >90% of the genome meeting minimum quality thresholds.

Variant calling for single-nucleotide variants (SNVs), small insertions/deletions (indels), and copy number variants (CNVs) was performed using the DRAGEN pipeline. CNV detection utilized a reference-based normalization algorithm with matched normal samples. Variant annotation incorporated multiple databases, including Online Mendelian Inheritance in Man (OMIM), GenBank, Database of Single Nucleotide Polymorphisms (dbSNP), 1000 Genomes, Human Gene Mutation Database (HGMD), ClinVar, VarSome, and an internal database enriched for Arab population variants. Variants were classified according to American College of Medical Genetics and Genomics/Association for Molecular Pathology (ACMG/AMP) guidelines, and variants of interest were confirmed by Sanger sequencing.

Analysis was performed using a phenotype-driven approach focusing on genes relevant to autoinflammatory, immunodeficiency, and immune dysregulation syndromes. Variants were filtered based on sequencing quality metrics, population allele frequency, predicted functional impact, inheritance pattern, and clinical relevance. No additional candidate variants of clinical significance were identified beyond the reported heterozygous STING1 c.841C>T (p.Arg281Trp) variant.

The diagnosis was established approximately three months after presentation, during which time the patient had already fulfilled the criteria for chronic kidney disease requiring ongoing renal replacement therapy.

The patient continued corticosteroid therapy, resulting in gradual normalization of liver function over approximately three months, and is currently being evaluated for renal transplantation. Upon trial corticosteroid withdrawal, the patient’s liver function deteriorated with no evidence of new viral activation; therefore, maintenance corticosteroid therapy was resumed.

Genetic counseling and family screening were performed following the identification of the STING1 variant. Paternal Sanger sequencing confirmed that the heterozygous STING1 (c.841C>T; p.Arg281Trp) variant was inherited from the father, who remained asymptomatic.

## Discussion

This case represents an unusual intersection between a viral infection and genetically mediated immune dysregulation. An 18-year-old apparently immunocompetent adolescent with no family history of autoimmune, immunodeficiency, or genetic disorders presented with AKI and mixed cholestatic-hepatocellular liver injury following CMV infection. Genetic testing revealed a heterozygous STING1 mutation (c.841C>T; p.Arg281Trp), inherited from her asymptomatic father. This genetic finding may have contributed to the exaggerated hyperinflammatory response observed following CMV infection and, to our knowledge, represents the first reported case of symptomatic disease associated with heterozygous p.Arg281Trp carriage, raising the possibility of incomplete penetrance, modifier effects, or environmental triggers.

The cyclic GMP-AMP synthase (cGAS)–STING pathway is a critical component of the innate immune system and is responsible for detecting cytosolic dsDNA during both pathogen invasion and host damage, thereby initiating a robust type I interferon (IFN) response (18, 19).

Upon recognition of cytosolic DNA, cGAS catalyzes the synthesis of the second messenger 2’3’-cyclic GMP-AMP (cGAMP), which binds to STING, an endoplasmic reticulum (ER)-resident adaptor protein (19).

This binding triggers STING translocation from the ER to the Golgi apparatus, where it activates downstream signaling through TANK-binding kinase 1 (TBK1) and interferon regulatory factor 3 (IRF3), ultimately inducing type I interferons and pro-inflammatory cytokines (3, 18).

SAVI is a monogenic type I interferonopathy caused by gain-of-function mutations in STING1 (previously TMEM173) (20). SAVI is characterized by early-onset systemic inflammation, cutaneous vasculopathy predominantly affecting acral and cold-sensitive areas, and progressive interstitial lung disease (ILD) (20). The disease results from constitutive, ligand-independent STING activation leading to chronic upregulation of type I IFN signaling (20). Although most SAVI cases present during infancy, late-onset presentations in adulthood have been documented, reflecting the phenotypic heterogeneity of STING1-related diseases (21).

Prior to the present case, the p.R281W variant was exclusively reported in the context of homozygous inheritance. Alghamdi et al. (2021) described this variant in two homozygous siblings, demonstrating that the variant segregated as homozygous in all affected individuals and as either heterozygous or wild-type in all healthy family members (18). Wan et al. (2022) subsequently analyzed 12 cases of the p.R281W variant, all of which occurred in the homozygous state, and confirmed that heterozygous carriers were healthy with normal interferon activation (19).

Our patient challenges this paradigm by demonstrating clinically significant disease despite being a heterozygous carrier. Variable penetrance has increasingly been recognized in STING1-related diseases. For instance, Kang et al. (2025) reported a case involving the p.R218Q variant inherited from an asymptomatic 32-year-old father (20).

Liu et al. (2019) identified polymorphisms in interferon induced with helicase C domain 1 (IFIH1) and sterile alpha motif and histidine-aspartate domain-containing protein 1 (SAMHD1), which modified the phenotypic spectrum in a family with a novel G207E STING mutation. (21) Ritchie et al. (2021) noted that while most patients with SAVI are symptomatic from infancy, late-onset in adulthood can occur (22).

We hypothesize that CMV infection may have served as a “second hit,” amplifying baseline STING hyperactivation and precipitating disease in our heterozygous p.R281W patient while remaining silent in her father. This theory remains speculative because of the lack of functional evidence in family members and the absence of additional cases reported in the literature (p.Arg281Trp in heterozygous patients). Modifier genes or epigenetic factors may also have influenced disease expression and sex-specific differences in immune regulation, contributing to differential penetrance.

The classical SAVI phenotype is characterized by pulmonary and cutaneous manifestations, with interstitial lung disease occurring in up to 100% of patients and skin vasculopathy in approximately 85% (23). In contrast, our patient presented with a predominantly renal-hepatic phenotype, namely, AKI with acute tubulointerstitial nephritis and mixed cholestatic-hepatocellular liver injury, but without pulmonary or cutaneous involvement. This presentation expands the phenotypic spectrum of STING1-related diseases. Although hepatic involvement has been reported in SAVI, it remains a rare manifestation. The 2021 EULAR/ACR guidelines note that the clinical data regarding the hepatic manifestations of SAVI remain limited; however, case reports have described necrotizing granulomatous and cholestatic hepatitis and cholangitis with multiple biliary cysts (24). Different SAVI phenotypes are illustrated in **Table 2**.

**Table 2:** Pathogenic STING1 variants and associated phenotypes.

Variant	Inheritance	Clinical phenotype	Severity	Key features	Reference
p.V155M (V154M)	AD	Classic SAVI	Severe	Most common SAVI variant: ILD (100%), skin vasculopathy (86%), systemic inflammation (90%); end-stage respiratory failure in 6/21 by teenage years	(23)
p.N154S (N153S)	AD	Classic SAVI	Severe	Pulmonary fibrosis, combined innate/adaptive immunodeficiency, vulnerability to gammaherpesvirus infection	(30)
p.V147L	AD	Classic SAVI	Severe	Early-onset ILD, skin vasculopathy, systemic inflammation	(23)
p.V147M	AD	Classic SAVI	Severe	Similar to the V155M phenotype	(23)
p.R218Q	AD	Variable/Incomplete penetrance	Mild-severe	ILD without cutaneous features; asymptomatic carriers reported (32-year-old father)	(20)
p.R281W	AR (homozygous)	Recessive SAVI	Mild-moderate	ILD in all; cutaneous vasculitis in only 1/4; heterozygous carriers are healthy	(18)
p.R281Q	AD	SAVI	Moderate-severe	Defines novel functional cluster at positions 206, 281, 284	(3)
p.R284G	AD	SAVI	Severe	Part of a functional cluster; constitutive TBK1-mediated activation	(3)
p.R284W	AR (requires homozygosity)	Recessive SAVI	Variable	Only the additive STING1 GOF mutation requires homozygosity	(24)
p.C206Y	AD	SAVI	Moderate-severe	Novel cluster; constitutive IFN activation	(3)
p.F269S	AD	SAVI	Severe	Novel variant; endothelial toxicity, ligand-independent Golgi translocation, naïve T cell predominance	(1)
p.N188H	AD	SLE-like (atypical)	Moderate	Novel variant; childhood-onset SLE without typical SAVI features; elevated ISGs	(31)

AD, autosomal dominant; AR, autosomal recessive; SLE, systemic lupus erythematosus; ILD, interstitial lung disease; GOF, gain-of-function; IFN, interferon; ISGs, interferon-stimulated genes; SAVI, STING-associated vasculopathy with onset in infancy; STING1, stimulator of interferon response CGAS Complex 1; TBK1, TANK-binding kinase 1.

The mixed cholestatic-hepatocellular injury pattern presented in the patient, with zone 3 hepatocellular and canalicular cholestasis revealed on biopsy, represents a distinct hepatic phenotype not previously associated with the p.R281W variant. Emerging evidence indicates that the cGAS–STING pathway plays a significant role in liver pathophysiology, with its aberrant activation contributing to hepatic inflammation and injury in multiple liver diseases (25, 26).

Renal involvement in SAVI is under-recognized. However, recent studies have linked aberrant STING activation to various kidney disorders, including AKI, microvascular affection, podocytopathies, and tubulointerstitial disease (27, 28). Volpi et al. (2019) reported hematuria resolution in a patient with SAVI treated with ruxolitinib, suggesting that renal manifestations may be part of the SAVI spectrum (16). Our patient’s biopsy findings of acute tubular injury with mild interstitial nephritis and focal tubulitis were consistent with those of STING-mediated renal inflammation. Notably, STING has been shown to promote ferroptosis and tubular injury in AKI models through nuclear receptor coactivator 4 (NCOA4)-dependent ferritinophagy (29).

The absence of pulmonary and cutaneous manifestations in our patient, despite the presence of a pathogenic STING1 variant, underscores the phenotypic heterogeneity of STING1-related diseases. This variability may reflect differences in tissue-specific STING expression, modifier genes, or environmental triggers.

Concurrent CMV viremia (17,000 copies/mL) in our patient raised the question of whether the viral infection served as a trigger that unmasked the phenotype in the context of heterozygous STING1 carriage. CMV activates the cGAS–STING pathway and induces robust type I IFN responses (32, 33). Lio et al. (2016) demonstrated that STING is essential for the initial detection of CMV and for the first wave of interferon production, which controls early viral replication (33). Furthermore, Baruah et al. (2025) showed that the Bruton’s tyrosine kinase (BTK)- *DEAD-box helicase 41* (DDX41)-STING signaling axis is activated during CMV lytic infection, highlighting the importance of this pathway in anti-CMV immunity (34).

We hypothesize that CMV infection may have acted as one potential environmental trigger contributing to disease expression in this genetically predisposed patient; however, alternative infectious, environmental, stochastic, or genetic modifying factors cannot be excluded. This hypothesis is supported by several observations. First, CMV activates the same cGAS–STING pathway that is constitutively hyperactive in STING1 gain-of-function carriers, potentially creating a synergistic inflammatory response (32, 33). Second, the patient’s father, who carried the same heterozygous variant, remained asymptomatic, suggesting that heterozygous carriage alone may be insufficient to cause the disease without an additional trigger.

Broader research has indicated that defects in the interferon pathway are linked to clinically significant CMV infections (33). Our case suggests that CMV infection may serve as an environmental trigger capable of unmasking STING1-related diseases in heterozygous carriers. In our patient, functional assays, including IFN signature measurements, were not performed due to unavailability; moreover, the patient’s clinical circumstances could have confounded their interpretation even if available. Glucocorticoid therapy was initiated before the genetic testing results were available due to the severity of the presentation. The 2021 EULAR/ACR guidelines explicitly state that IFN signature scores “may be negative in the diagnostic phase in patients with milder disease, or in response to glucocorticoid treatment.” (24).

Furthermore, concurrent CMV infection could confound the interpretation, as CMV itself activates the cGAS–STING pathway and induces interferon responses (33, 34). The 2021 EULAR/ACR guidelines acknowledge that “a practical barrier is the limited number of centers with the ability to check an IFN signature” and that “a chronically elevated peripheral blood IFN signature is not required for diagnosis.” (24) A published case series involving the p.R281W variant relied on this original functional characterization rather than performing *de novo* functional assays in certain cases (19). The gain-of-function nature of the STING1 p.R281W variant was established by Melki et al. (2017) through comprehensive *in vitro* functional characterization (3), and structural analysis confirmed that positions 206, 281, and 284 define a novel cluster of amino acids with functional importance in STING regulation (28).

Despite the absence of functional assays, the available clinical and immunologic findings suggest that the variant may have contributed to disease susceptibility and immune dysregulation. The persistent inverted CD4/CD8 ratio, even after viral clearance, is consistent with T-cell defects documented in patients with SAVI (23) and suggests ongoing immune dysregulation independent of active infection. The patient’s steroid dependence, with clinical deterioration upon steroid withdrawal despite viral clearance, further supports an underlying steroid-dependent autoinflammatory process rather than a CMV-driven disease. Negative CMV immunostaining in both the renal and hepatic tissues excluded direct viral cytopathic injury, whereas the histopathological findings of acute interstitial nephritis and zone 3 cholestasis were consistent with immune-mediated pathology. Finally, the disproportionate hyperinflammatory response to CMV infection, with a marked elevation of inflammatory markers and multi-organ involvement, was atypical for CMV infection in an immunocompetent host and suggested an underlying predisposition. Collectively, these observations serve as clinical functional assays to demonstrate the pathogenic consequences of STING1 variants *in vivo*.

Although our patient improved with antiviral therapy and corticosteroids and did not require JAK inhibitor treatment, JAK inhibition has been reported as a therapeutic strategy in selected patients with STING1-associated interferonopathies. In similar refractory or steroid-dependent cases, it may represent a potential future therapeutic option; however, this remains speculative in our case. This approach targets the pathogenic interferon signaling pathway directly while potentially allowing steroid tapering. However, monitoring for viral reactivation is essential, as BK virus and herpes reactivation have been reported in patients with interferonopathy treated with JAK inhibitors (24).

## Limitations

This case report has several important limitations. Functional assays, including IFN-β luciferase reporter activity and TBK1/IRF3 phosphorylation studies, were not performed because of resource constraints, concurrent glucocorticoid therapy, and active CMV infection, all of which could have confounded the interpretation of the results. Consequently, a definitive causal relationship cannot be established in the absence of functional validation.

The findings suggest that the heterozygous STING1 p.Arg281Trp variant may act as a potential predisposing factor for CMV-triggered HLH-like hyperinflammatory disease. However, this observation should be regarded as hypothesis-generating given the lack of functional validation, absence of interferon signature testing, inability to assess subclinical pathway activation in family members, and the inherent limitations of a single-case report. Future studies involving functional assays, segregation analyses, and additional reported cases will be necessary to better define the penetrance, pathogenicity, and mechanistic role of this variant.

Although paternal inheritance of the variant raises the possibility of incomplete penetrance, functional studies in family members could not be performed to determine whether asymptomatic carriers might exhibit subclinical interferon pathway activation. Furthermore, although phenotype-driven whole-exome sequencing did not identify additional clinically significant variants, the contribution of unidentified genetic modifiers cannot be completely excluded.

Finally, although the patient exhibited multiple clinical and laboratory features suggestive of hemophagocytic lymphohistiocytosis, a definitive diagnosis of HLH could not be confirmed because the HLH-2004 diagnostic criteria were not fully met, and bone marrow evaluation was not performed.

## Conclusion

Our findings reveal an unrecognized inheritance pattern of the p.Arg281Trp variant and an atypical clinical presentation, thereby expanding the known clinical disease spectrum and challenging existing assumptions. Although this variant has been previously reported, its clinical behavior in this specific context provides novel insights into the underlying disease biology and may facilitate earlier and more accurate diagnosis. This case illustrates how a genetically primed immune system can convert a viral infection into an HLH-like inflammatory syndrome, underscoring the value of genomic evaluation in unexplained hyperinflammation and supporting pathway-based personalized management strategies for interferon-mediated immune disorders.

## Data Availability

The raw data supporting the conclusions of this article will be made available by the authors, without undue reservation.

## Glossary

ACMG/AMP: American College of Medical Genetics and Genomics/Association for Molecular Pathology
ACR: Albumin-creatinine ratio
AKI: Acute kidney injury
ALP: Alkaline phosphatase
ALT: Alanine aminotransferase
AMA: Anti-mitochondrial antibody
ANA: Antinuclear antibody
ANCA: Antineutrophil cytoplasmic antibodies
ASMA: Anti-smooth muscle antibody
AST: Aspartate aminotransferase
anti-CCP: Anti-cyclic citrullinated peptide
anti-dsDNA: Anti-double stranded DNA
anti-GBM: Anti-glomerular basement membrane
anti-Scl-70: Anti-topoisomerase I
anti-SSA/Ro: Anti-Sjögren’s syndrome-related antigen A
anti-SSB/La: Anti-Sjögren’s syndrome-related antigen B
BTK: Bruton’s tyrosine kinase
cGAMP: Cyclic GMP–AMP
cGAS: Cyclic GMP–AMP synthase
C3,C4: Complement 3 and 4
CD: Cluster of differentiation
CMV: Cytomegalovirus
CNVs: Copy number variants
COVID-19: Coronavirus disease 2019
CRP: C-reactive protein
dbSNP: Database of Single Nucleotide Polymorphisms
DDX41: DEAD-box helicase 41
DRAGEN: Dynamic Read Analysis for GENomics
EBV: Epstein–Barr virus
ER: Endoplasmic Reticulum
GRCh38: Genome Reference Consortium Human Build 38
H&E: Hematoxylin and eosin
HAV: Hepatitis A virus
HBV: Hepatitis B virus
HCV: Hepatitis C virus
HEV: Hepatitis E virus
HFE: Hemochromatosis gene
HGMD: Human Gene Mutation Database
HIV: Human immunodeficiency virus
HLH: Hemophagocytic lymphohistiocytosis
IFN: Interferon
IFIH1: Interferon-induced with helicase C domain 1
Ig: Immunoglobulin
ILD: Interstitial lung disease
Indels: Insertions/deletions
IRF3: Interferon regulatory factor 3
ISG: Interferon-stimulated gene
JAK: Janus kinase
LDH: Lactate dehydrogenase
LKM: Liver kidney microsomal antibody
MAVS: Mitochondrial antiviral-signaling protein
MRCP: Magnetic resonance cholangiopancreatography
NCOA4: Nuclear receptor coactivator 4
OMIM: Online Mendelian Inheritance in Man
PCR: Polymerase chain reaction
PET-CT: Positron emission tomography-computed tomography
RF: Rheumatoid factor
SAVI: STING-associated vasculopathy with onset in infancy
SAMHD1: Sterile alpha motif and histidine–aspartate domain–containing protein 1
sCD25: Soluble CD25
sIL-2R: Soluble interleukin-2 receptor
SNVs: Single-nucleotide variants
STING: Stimulator of interferon genes
TBK1: TANK-binding kinase 1
TMEM173: Transmembrane protein 173
